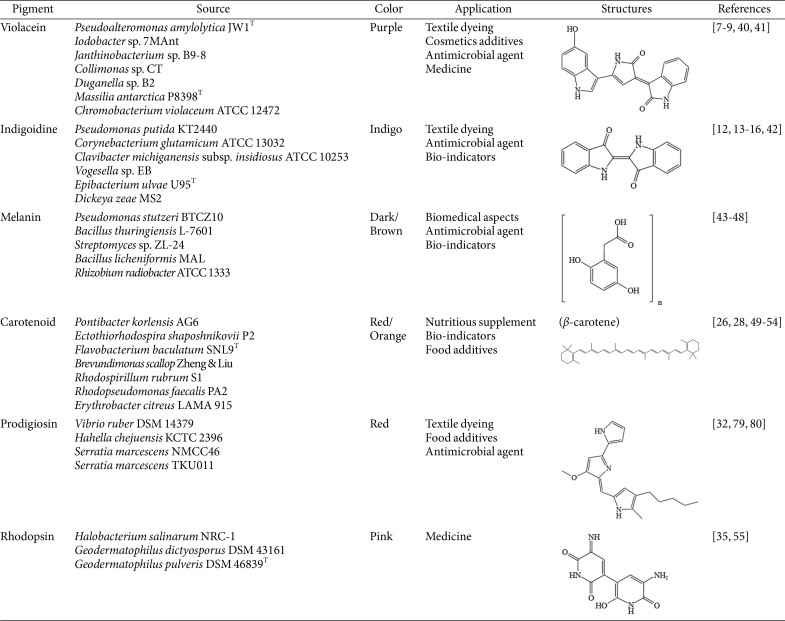# Bacterial Pigments as a Promising Alternative to Synthetic Colorants: From Fundamentals to Applications

**DOI:** 10.4014/jmb.2404.04018

**Published:** 2024-09-11

**Authors:** Xin Huang, Longzhan Gan, Zhicheng He, Guangyang Jiang, Tengxia He

**Affiliations:** 1 Key Laboratory of Plant Resource Conservation and Germplasm Innovation in Mountainous Region (Ministry of Education), College of Life Sciences, Guizhou University, Guiyang 550025, Guizhou Province, P.R. China; 2 Key Laboratory of Leather Chemistry and Engineering (Ministry of Education), College of Biomass Science and Engineering, Sichuan University, Chengdu 610065, Sichuan Province, P.R. China

**Keywords:** Bacterial pigments, pigment classification, biosynthesis, chemical structure, biological activity

## Abstract

Pigments find widespread application in the fields of food, medicine, textiles, and cosmetics. At present, synthetic colorants dominate the global pigment market. However, the environmental and health hazards associated with synthetic colorants have spurred extensive research on eco-friendly and safe alternatives. Natural pigments are particularly intriguing for meeting consumer demands and sustainable development, as they not only exhibit various vibrant color shades without discernible toxic side effects but also offer additional healthful features such as antibacterial, antioxidant, anticancer, and antiviral properties compared with their synthetic counterparts. Among natural sources, bacterial strains share distinct advantages for large-scale pigment production because of their intrinsic robustness of cellular metabolic systems. This review comprehensively outlines the bacterial sources, extraction and purification methods, structural characteristics, biological activities, and potential applications of typical pigments, including but not limited to violacein, indigoidine, melanin, carotenoids, prodigiosin, and rhodopsin. Additionally, it underscores the primary obstacles to the development and production of bacterial pigments for commercial applications, discussing feasible strategies for overcoming production bottlenecks. This work also provides valuable insights for the scientific and rational advancement of bacterial pigment development.

## Introduction

Pigments, defined as chemical compounds that impart color to a substance, have a long history of use. Initially, simple techniques involved rubbing crushed pigments onto fabrics. Over time, methods for extracting pigments from plants were developed, leading to the discovery of various dyes [1], such as carminic acid and red yeast rice. With the progress of civilization, the practice of dyeing expanded, and by the 4th century, additional pigments such as indigo were uncovered. Natural pigments were widely utilized and traded, serving as a significant source of global wealth until the advent of synthetic dyes in 1856 by Perkin. Synthetic coloring agents quickly gained popularity due to their convenient synthesis, superior coloring properties, affordability, and high stability, causing a decline in the use of natural pigments [2]. Presently, synthetic pigments have diverse applications across fields such as the textile industry, food processing, agricultural research, electronics, and cosmetics. However, most of the synthetic pigments are non-degradable, posing a considerable risk of environmental pollution. Furthermore, some synthetic pigments exhibit toxicity and carcinogenic properties, which can harm human health [3]. The shift toward naturally derived alternatives has been fueled by consumer perceptions and demands. Today, synthetic additives are considered "toxic contaminants," leading consumers to express reluctance toward their use. An increasing number of individuals advocate for the utilization of natural pigments, marking a global inclination toward their preference.

Natural colorants have garnered increased attention due to their non-toxic, non-carcinogenic, and biodegradable properties. Materials for natural dyeing encompass ores, insects, plants, and microorganisms. However, cultivating plants exclusively for dyeing purposes may incur high costs. The production of plant-based pigments is hindered by a prolonged lead time and unpredictable nature, posing challenges for batch reproducibility. Large-scale plant use may lead to the depletion of valuable species. Consequently, researchers have explored an alternative strategy involving microorganisms.

Unlike other sources, bacterial strains offer advantages such as a short life cycle, independence from seasonal constraints, ease of downstream processing, and the ability to produce pigments in various colors and shades. Thus, bacterial pigments are highly attractive for the developers when compared with synthetic colorants as well as plant pigments. The production processes for bacterial pigments involving fermentation, extraction, purification and desiccation steps greatly strengthen their feasibility. More importantly, it's reported that certain bacterial strains are capable of utilizing various agro-industrial wastes (*e.g.*, molasses, corncobs, sugarcane bagasse, wheat straw, and peels) as fermentation substrates for pigment biosynthesis [4], meaning that a promising alternative strategy for cost-effective and sustainable production of bacterial pigments.

**Fig. 1:**
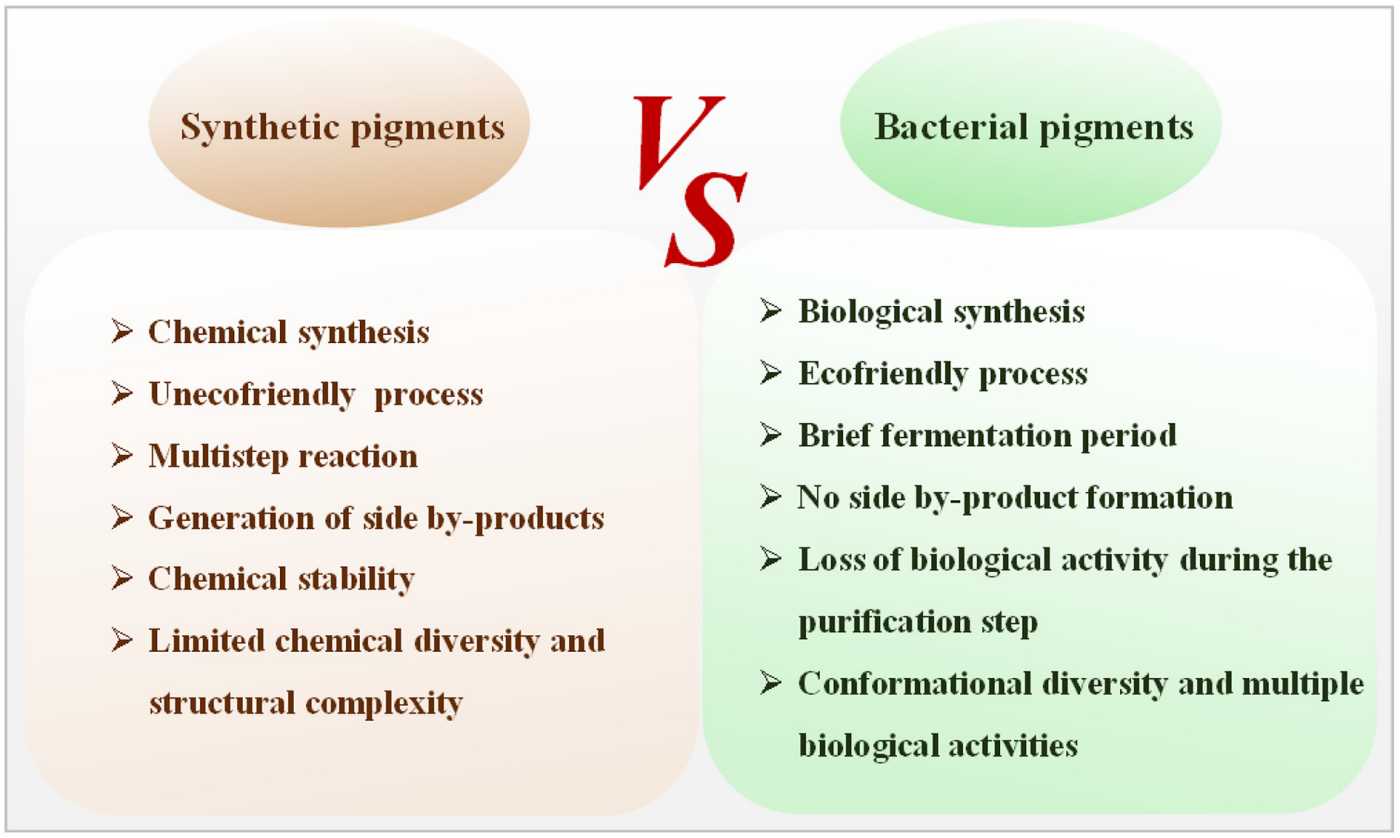
Multi-aspect comparisons between synthetic and bacterial pigments.

Bacteria demonstrate a remarkable ability to produce a diverse range of natural pigments, crucial for their adaptation to extreme environmental conditions and the execution of specific cellular functions. These pigments not only are environmentally friendly and safe for human health but also possess significant pharmacological activities, encompassing anti-inflammatory, anti-allergic, antineoplastic, anticancer, and antioxidative properties. These pigments therefore exhibit extensive potential applications across various industries including textiles, food, cosmetics, and pharmaceuticals. Currently, a limited set of bacterial pigments, such as indigo, riboflavin, *β*-carotene, lycopene, astaxanthin, and monascus pigments, have garnered approval from regulatory authorities such as the United States Food and Drug Administration and the European Food Safety Authority. It's estimated that the market value of pigments will be expected to reach US$33.2–49.1 billion by 2027 [4]. Carotenoids, known for their numerous health benefits, are highly valued pigments in commercial applications. In 2020, the market size for carotenoids was US$1.7 billion, with a projected global market reaching US$2 billion by 2027 [5]. Currently, carotenoid synthesis primarily relies on chemical methods due to the comparatively lower costs of synthetic carotenoids ($250–2,000 kg^−1^) compared to their natural counterparts [6]. However, natural carotenoids command higher market values ranging from $350 to $7,500 kg^−1^. The production of natural carotenoids using bacteria has emerged as a notable research focus due to the high costs associated with plant-derived carotenoids. Overall, the utilization of natural coloring agents has gained extensive interest worldwide, which is expected to further stimulate the coloring market. A comparison of synthetic and bacterial pigments is shown in Fig. 1.

This review systematically describes natural resources, structures, and biological activities of pigments, covering extraction, separation, and characterization. Emphasis is placed on applications of bacterial pigments, addressing existing challenges, and proposing engineering strategies for bacterial synthesis. The study serves as a reference to promote the application of bacterial pigments and advocate for the replacement of synthetic pigments with natural alternatives.

## Typical Pigments of Bacterial Origin

### Violacein

Violacein is a natural indolocarbazole compound that presents as purple. It is formed by the condensation of two tryptophan molecules. Initially isolated from *Chromobacterium violaceum*, this pigment exhibits maximum light absorption at 575 nm. Various bacteria from diverse genera, including *Pseudoalteromonas*, *Iodobacter*, *Janthinobacterium*, *Collimonas*, *Duganella*, and *Massilia* [7-9], can produce violacein, as tabulated in Table 1. The octanol-water partitioning coefficient for violacein is 3.34, indicating high hydrophobicity and suggesting that the compound is not easily released by the host into the surrounding environment. Currently, extensive research has focused on the biosynthesis and biological properties of violacein. This violet pigment is often associated with biofilm formation, and its synthesis occurs through quorum sensing mechanisms, making violacein-synthesizing bacteria an ideal model for investigating the effect of various molecules on quorum sensing. Researchers harnessed the *C. violaceum* strain to meticulously assess pigment production in the presence of nanoparticles, offering novel approaches to combat bacterial persistent infections and counter multidrug resistance and the associated challenges [8]. As a natural pigment, violacein exhibits diverse biological activities, including antibacterial, antiviral, antiulcerogenic, and anticancer properties with potential medical applications. All the known violacein-producing species are heterotrophic, predominantly found within aquatic ecosystems. Strains of the genus *Massilia*, obtained from freshwater samples collected in James Ross Island and Eagle Island in Antarctica, exhibit the ability to produce violacein, positioning them as promising candidates for natural pigment production [10]. Violacein confers a survival strategy for its producers, providing selective advantages against other bacterial cells and effective defense and deterrence against bacterivores including protozoa and nematodes [11].

### Indigoidine

The indigo dye is a pioneering blue pigment that has widespread application, particularly in textile dyeing. This pigment is the result of the condensation of two L-glutamine molecules, orchestrated by the nonribosomal peptide synthetase (NRPS) enzyme, which plays a pivotal role in its synthesis (Table 1). The stability of both intermolecular and intramolecular hydrogen bonds in this compound contributes to key properties, including a high melting point (390–392°C), ensuring indigós stability as a dye [12]. Furthermore, indigo exhibits limited solubility in organic solvents and remains insoluble in water. Several bacteria, initially identified in *Pseudomonas indigofera*, and later in genera such as *Arthrobacter*, *Erwinia*, *Corynebacterium*, *Clavibacter*, *Vogesella*, *Phaeobacter*, *Photorhabdus*, *Dickeya*, and *Streptomyces*, have the capacity to produce this pigment [2]. Day and his team discovered that microbes producing indigoidine may gain a competitive advantage due to its antioxidant activities, mainly through the production of reactive oxygen species, and antimicrobial activities, mainly by inhibiting colonization of competing microorganisms in the environment [13-15]. Additionally, Cude *et al*. reported that indigo from *Phaeobacter* sp. strain Y4I has inhibitory effects on *Vibrio fischeri*, correlating with the degree of pigmentation [16]. Current efforts are directed toward addressing low pigment yield in industrial applications. Ghiffary *et al*. reported the successful production of indigoidine at a high concentration and with remarkable productivity using a metabolically engineered strain of *Corynebacterium glutamicum*. The final indigoidine yield reached an impressive 49.30 g/l, achieving a productivity rate of 0.96 g/l/h [12]. This breakthrough underscores the potential for sustainable pigment production. The presence of functional groups, such as carboxyl and amine, in the indigoidine molecule also opens up possibilities for its application in supercapacitors and batteries. Notably, indigo-based chemosensors have been realized, including Fereja *et al*.'s chemiluminescence system utilizing indigo carmine/glucose/hemin/H_2_O_2_ for glucose determination in blood and urine, showcing medical applications [17].

### Melanin

Melanins constitute a heterogeneous and polymeric group of pigments. Primarily formed through the conversion of tyrosine (DOPA-pathway) or malonyl-coenzyme A (DHN-pathway), these pathways are prevalent in bacteria, as detailed in Table 1. With higher molecular weight, melanin is found in fossils, hair, skin, scales, plants, marine cephalopods, bacteria, and fungi [18]. Exhibiting an amorphous nature, melanin typically appears dark brown to black, occasionally displaying red and yellow hues. Its exceptional photochemical stability translates to insolubility in both organic and aqueous solvents and resistance to high temperatures, even up to 600°C. Melanin can be categorized into five groups based on precursor chemical properties: eumelanin, pheomelanin, neuromelanin, allomelanin, and pyomelanin. This pigment is present in various species, including *Rhizobium* sp., *Bacillus thuringiensis*, *Pseudomonas aeruginosa*, *Burkholderia cenocepacia*, and *Vibrio nigripulchritudo* and has demonstrated excellent biocompatibility and biostability, as evident from the absence of side effects associated with cytotoxicity and antigenic reactions in living organisms [19]. Melanin has a relatively high half-life because of the lack of enzymes degrading these pigments in living cells. The genus *Streptomyces* is widely studied as a source of antibiotics, and this bacterium is identified the production of melanin-type pigments [20]. The biological activity of melanin is intricately tied to its structure, providing photoprotective functions against radiation in organisms owing to its highly conjugated structures, effectively shielding cell from radiation-induced damage. Research has highlighted melanin's capabilities in scavenging free radicals, chelating metal ions, and exhibiting antioxidant and antibacterial activities. Beyond its applications in agriculture and food production, melanin's potential extends to various industries. Recent studies have explored its antitumor, immune regulation, radiation protection, and photothermal properties. In biomedical contexts, melanin has been applied as magnetic resonance imaging contrast agents, photothermal agents, and medical antitumor materials. Notably, research on melanin's in vitro neurotoxicity and neuroprotective effects has gained extensive attention, particularly in the exploration of Parkinson's disease, Alzheimer's disease, and various neurological conditions in clinical settings [21, 22].

### Carotenoids

Carotenoids, vital lipid-soluble pigments categorized as isoprenoid-derived natural products, play a crucial role in various organisms, ranging from microorganisms to plants. They are classified into four groups—C30, C40, C45, and C50—based on the number of carbons in their chemical structures (Table 1). Bacteria predominantly synthesize C30 and C40 carotenoids through two pathways, namely the mevalonate pathway and the 2-C-methyl-D-erythritol-4-phosphate pathway, catalyzed by specific enzymes [23].

The maximum absorption range for carotenoids spans from 440 to 520 nm. Depending on their structure, carotenoids can exhibit colors ranging from yellow to deep red. To date, more than 700 carotenoid varieties have been identified [24]. Among these, *β*-carotene is highly sought after, featuring two *β*-ionic rings that can break down into two retinol molecules, making it a pro-vitamin A. Notably, *β*-carotene contributes to safeguarding eyesight and enhancing eye health. Carotenoids are indispensable for the survival of photosynthetic organisms, usually associating with photosynthetic membranes and non-covalently binding to specific pigment-protein complexes. Their primary function is to absorb and transfer light energy, providing photoprotection as a co-factor in photosynthesis. Beyond their role in photosynthesis, carotenoids offer numerous health benefits, such as boosting the immune response, preventing cancer, and serving as antioxidants and anti-inflammatories. A diet rich in vegetables and fruits, abundant sources of carotenoids, may potentially fortify the immune system and reduce the risk of degenerative diseases, including Alzheimer’s disease and Parkinson's disease. Carotenoids also play a vital role in safeguarding microbial cells from photo-oxidative injury and environmental stresses at low temperatures. These pigments are able to efficiently respond to a lower temperature and freeze-thaw cycles [25]. Notably, astaxanthin, which is derived from the strain *Pontibacter korlensi* AG6 and is one of the most promising subclasses in biomedical applications, has shown antibacterial, antioxidant, and cytotoxic properties in breast cancer cell lines [26]. Another carotenoid, zeaxanthin, containing oxygen, is considered a safe food additive and can be incorporated into animal feed [27]. Studies on marine photosynthetic bacteria reveal carotenoids' suitability for storage in dark, low-temperature, and neutral-to-alkali conditions. In the presence of metals such as Na^+^, Mg^2+^, and Fe^3+^, carotenoids show varying degrees of decrease [28].

### Prodigiosin

Prodigiosin, a red pigment with a pyrrolylpyrromethane skeleton, is synthesized by the condensation of two key intermediates, namely 2-methyl-3-n-amylpyrrole and 4-methoxy-2-2'-bipyrrole-5-carbaldehyde (Table 1). This pigment is primarily produced by *Serratia marcescens*, a gram-negative bacterium belonging to the family Enterobacteriaceae. This ubiquitous bacterium has a tendency to produce various pigmented colonies that often contain prodigiosin [29]. Prodigiosin appears only in the later stages of bacterial growth, known as the idiophase, and its biosynthesis is regulated by pheromone-mediated transcription and controlled through quorum sensing. This pigment is also produced by other genera including *Streptomyces*, *Vibrio*, *Hahella*, and *Zooshikella* [30]. Prodigiosin exhibits significant antimicrobial activity, with distinct mechanisms of biological action against various bacteria, including outer membrane damage, disrupted cellular respiration, and inhibited RNA and protein synthesis [31]. Notably, prodigiosin demonstrates enhanced antibacterial efficacy against gram-positive bacteria, including *Staphylococcus aureus* and *B. subtilis*, when compared with its activity against gram-negative bacteria. Prodigiosin has broad-spectrum pharmaceutical properties, including antibacterial, anticancer, antimalarial, antidiabetic, antifungal, and antiprotozoal activities and immune system modulation [11]. It demonstrates enhanced antibacterial efficacy against gram-positive bacteria compared with gram-negative bacteria. Prodigiosin even exhibits inhibitory effects on *Borrelia burgdorferi*, the causative agent of Lyme disease [32]. Prodigiosin is one of the most promising bacteria-derived pigments. Although some biological mechanisms of this pigment are poorly described, the production of this pigment will continue to remain a hotspot topic. Future research will continue to focus on optimization of the production mode of this pigment and expand its applications in medicine.

### Rhodopsin

Rhodopsin, a photoreceptive protein containing a retinal chromophore, is not limited to animals but is also present in various microorganisms, and one typical structure is illustrated in Table 1. The retinal chromophore molecule absorbs light, thereby imparting red color. In vertebrates, rhodopsin mediates vision under low-light conditions and serves various functions in microorganisms. For instance, *Halobacterium halobium*, a type of Archaea, contains halorhodopsin that functions as a sodium pump mediated by luminescence [33]. Decades later, researchers identified new microbial rhodopsin in *H. halobium*, utilizing luminescence as an energy source for transmembrane ion flux, complementing the primary function of halorhodopsin [34]. Recent years have seen the discovery of diverse rhodopsins in microorganisms, revealing novel functions such as phototaxis and photomobility. Researchers frequently employ rhodopsin as a model to understand active membrane transport mechanisms and signaling sensors. Bacteriorhodopsins, for example, can regulate cellular behavior through proton-exchange, influencing a range of cell activities [35]. This approach is also used to reprogram human fibroblasts into neural cells, potentially enhancing neural regeneration [36]. In recent years, optogenetics technology has rapidly advanced, with microbial rhodopsin serving as a powerful tool for studying various nervous system diseases.

### Other Pigments

Some bacteria can synthesize specialized pigments as secondary metabolites, often linked to their pathogenicity or adaptation to extreme environments. Beyond typical pigments, additional pigments with unique biological functions hold significant potential for market development.

Pyocyanins and pyoverdines, produced by *P. aeruginosa*, play a crucial role in pathogenesis, intricately involved in iron metabolism. Pyocyanin, a greenish-blue pigment, participates in reduction mechanisms and releases iron from transferrins [37]. Pyoverdine, a greenish-yellow pigment, insulates iron from the environment and removes it from the host’s iron transport proteins, transferrins and lactoferrins [38]. Research on these pigments has focused particularly on the development of pigment-based therapies that can inhibit the production of virulence factors, mainly to effectively mitigate *P. aeruginosa* infections in hospitalized patients.

Flavins, characterized by a yellow pigment, feature riboflavin as the predominant microbial pigment, also known as vitamin B2. Riboflavin biosynthesis is a complex process involving a series of enzymatic reactions. Riboflavin serves as a structural component of coenzymes, participating in various cellular activities and playing key roles. Roseoflavin and toxoflavin, which are structural riboflavin-analogs isolated from *Streptomyces* spp. and *Burkholderia* spp., respectively, exhibit antimicrobial activity [39]. In summary, bacteria serve as an excellent natural source for pigment production, showing a diverse array of pigments with extensive applications.

## General Extraction and Separation Methods of Bacterial Pigments

The extraction methods of bacterial pigments depend on the characteristics of the target metabolite and its location within the culture. Microbial fermentation can result in the production of natural pigments, which can be secreted in two distinct ways: intracellularly and extracellularly. Traditional pigment extraction methods include solvent extraction, distillation, Soxhlet extraction, and maceration [56]. However, these methods come with several limitations, including high solvent consumption, lengthy extraction times, and low efficiency. Addressing the challenge of introducing cost-effective, efficient, and safe extraction techniques for natural pigment recovery is essential for enabling large-scale production.

### Extraction of Extracellular Pigments

Certain extracellular pigments are released into the fermentation solution. Modification of growth conditions, such as medium composition and process parameters, can significantly influence the properties and yield of these pigments. In the industrial sector, two types of fermentation techniques are widely employed. The first is the fed-batch approach, involving the targeted replenishment of fresh medium once one or more substrates have been depleted. Another technique is fed-batch fermentation, which supplies fresh nutrients to microbial cells, thereby delaying the exponential phase [57]. When coupled with suitable optimization processes in pigment production, these two technologies have the potential to yield substantial quantities of extracellular pigments that can be easily harvested or processed without resorting to solvent extraction. Presently, the use of aqueous two-phase systems has emerged as a promising method for extracting pigmented compounds from fermented broths. This approach utilizes liquid-liquid fractionation and relies on the application of environmentally friendly green ionic liquids (ILs) to extract pigmented molecules. This methodology not only ensures environmental sustainability but also offers significant economic benefits [58].

### Extraction of Intracellular Pigments

**Fig. 2:**
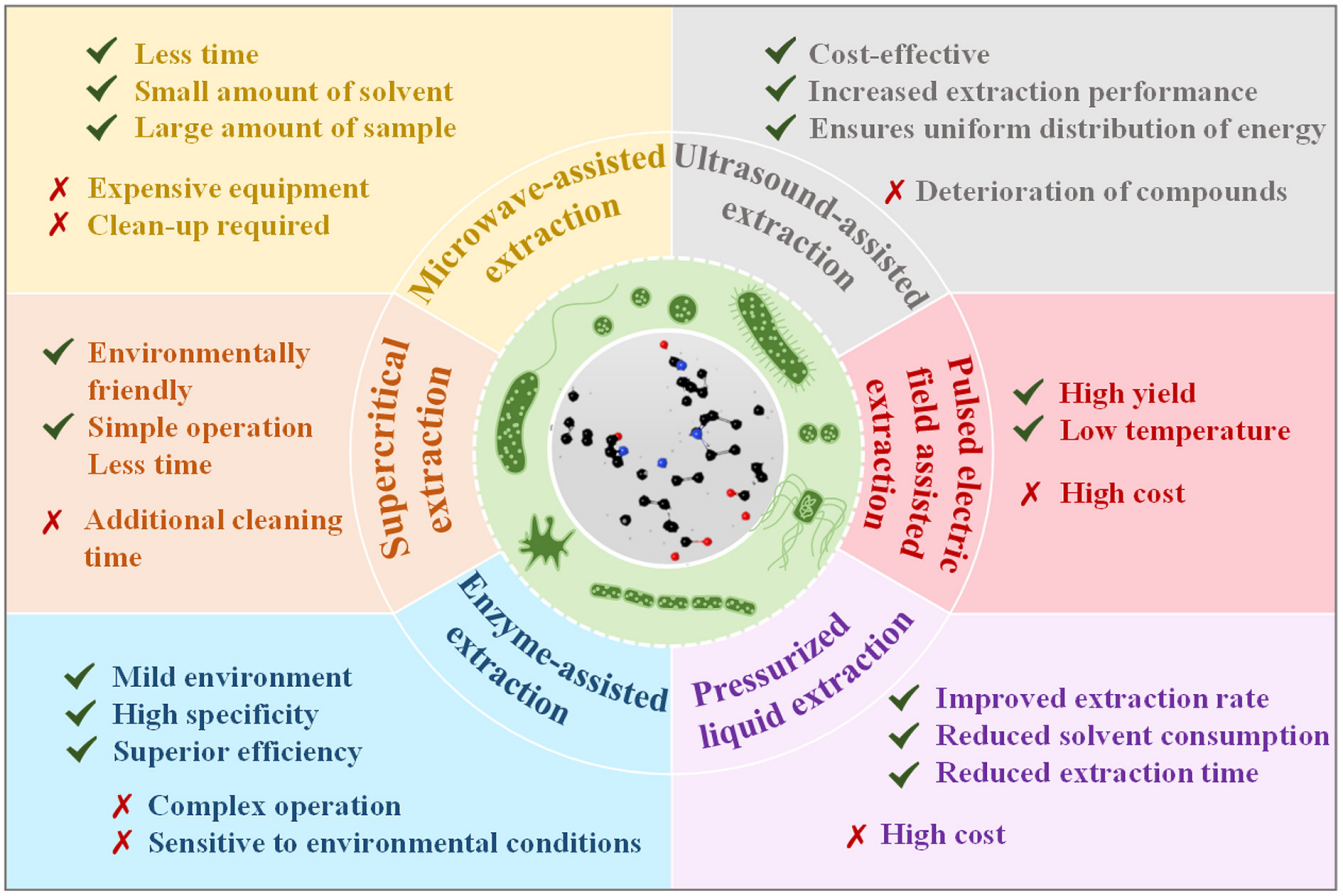
Advantages and disadvantages of various extraction methods for pigments from bacterial cells.

Certain bacteria can produce intracellular pigments, requiring specialized techniques for extraction from cellular structures. Fig. 2 shows advantages and disadvantages of several methods currently used for bacterial pigment extraction. These include microwave-assisted extraction, supercritical extraction, enzyme-assisted extraction, ultrasound-assisted extraction, pulsed electric field–assisted extraction, and pressurized liquid extraction [59].

Despite their advantages, these methods suffer from high costs and equipment inefficiency. To address these limitations, many studies have combined these extraction techniques. For example, Hasan *et al*. used ultrasonication with *β*-glucanase enzyme to extract astaxanthin from *Phaffia rhodozyma*, resulting in the most effective extraction method among all tested physical and chemical methods, yielding 435.71 ± 6.55 μg free astaxanthin per gram of yeast cell mass [60]. Rodrigues *et al*. innovatively combined UAE with ILs for phycobiliproteins extraction from spirulina (*Arthrospira*) platensis microalgae. Optimal results were achieved using 2-HEAA+2-HEAF as the solvent, operating at 25 kHz, pH 6.50, solvent: biomass ratio of 7.93 mg/l, and a 30-min extraction duration [61]. Hence, a variety of techniques should be employed for efficient pigment extraction.

## Characterization of Bacterial Pigments

To obtain highly pure pigments, additional steps involving the separation and purification of crude extracts are necessary. Commonly used techniques include column chromatography, membrane separation, ultrafiltration, and others. Raman spectroscopy and high-performance liquid chromatography (HPLC) are powerful tools for identifying bacterial pigments. Raman spectroscopy enables non-destructive pigment analysis by studying their vibration spectra, revealing structural characteristics. HPLC separates pigments based on their chemical properties, allowing precise identification and quantification of individual components [62, 63].

Moreover, for precise conformational and structural studies, modern techniques such as mass spectrometry (MS) are used, including liquid chromatography (LC)-MS, gas chromatography (GC)-MS, nuclear magnetic resonance spectroscopy, scanning electron microscopy, transmission electron microscopy, Fourier-transform infrared spectroscopy, electron spin resonance spectroscopy, and the combination of pyrolysis GC-MS (py-GC-MS) [64]. These techniques significantly advance our understanding of bacterial pigments, facilitating their application in various scientific fields such as food science, pharmaceuticals, and biotechnology.

## Multi-Perspective Applications of Bacterial Pigments

**Fig. 3:**
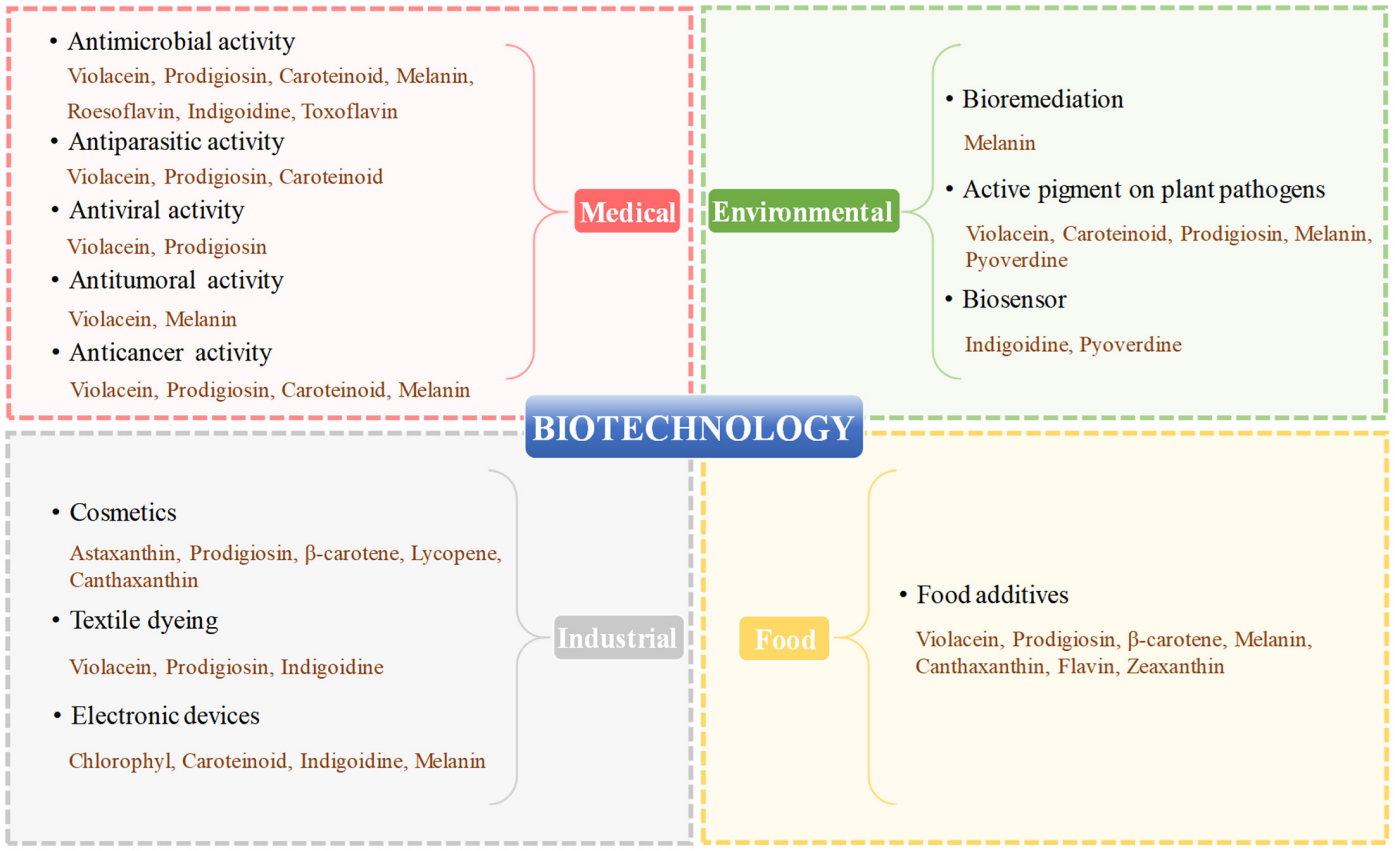
Biotechnological applications of a variety of bacterial pigments: medical, food, industrial and environmental biotechnology.

Pigments from a variety of bacterial strains exhibit a wide range of biotechnological activities, and they are widely utilized in the textile industry, agriculture, food industry and biomedical fields. In current years, bacterial pigments are facing a fast-growing global market and showing an overall upward trend in replacing synthetic ones [65]. The applications of bacterial pigments in various biotechnological fields are summarized in Fig. 3, and their general applications are outlined as follows.

### Bacterial Pigments in Medical Biotechnology

Medical biotechnology plays a crucial role in addressing challenges to human health, particularly in the face of threats such as the SARS-CoV-2 pandemic, increasing incidence of cancer, emergence of microbial superbugs, and increasing number of multidrug-resistant infections. The urgency of biomedical research is underscored by these issues. Bacterial pigments, known for their unique biological properties such as antibacterial, antitumor, antioxidant, anticancer, and antiviral activities, have garnered significant interest. The antimicrobial efficacy of violacein was first documented in 1942, when experiments were conducted by mixing the crude extracts of the violet pigment with a bacterial suspension, leading to the discovery that it effectively inhibited soil amoebas from ingesting the bacteria [66]. Combined application of violacein and *Bdellovibrio bacteriovorus* HD100 exhibited potential in controlling microflora within complex systems [67], including *S. aureus*, *Acinetobacter baumannii*, *Bacillus cereus*, and *Klebsiella pneumoniae*. Additionally, violacein has shown inhibitory effects on various cancer cells, including breast cancer and cervical HeLa cells [68, 69].

Significant progress has recently been achieved in researching pigments produced by microorganisms isolated from the ocean. Pachaiyappan *et al*. successfully isolated the marine endophytic bacterium *Pontibacter korlensi*s AG6, capable of producing astaxanthin. The study revealed that this pigment displays cytotoxic effects on the human breast cancer cell line (MCF-7), along with significant antibacterial and antioxidant activities [70]. In 2019, Abdelfattah *et al*. discovered prodigiosins derived from an actinomycete isolated from a marine sponge. This pigment exhibits antioxidant and anti-inflammatory effects, making it a potential drug target for preventing stomach damage and a possible alternative to the anti–gastric ulcer agent omeprazole [71]. Similarly, prodigiosin produced by *Streptomyces* sp. and *Zooshikella* sp. demonstrated effective antibacterial activity against *S. aureus* [72]. The C50 carotenoid bacterioruberin produced by the haloalkaliphilic archaeon *Natrialba* sp. M6 demonstrated anticancer properties and antiviral potency against hepatitis B and C viruses [73].

### Bacterial Pigments in Food Biotechnology

Food biotechnology aims to provide safer and healthier food options. Currently, the food industry heavily relies on synthetic pigments for coloration. While synthetic pigments offer advantages such as affordability, ease of production, and chemical stability, they also pose potential risks to human health, including allergenic, carcinogenic, and toxic properties. Conversely, natural counterparts avoid these unfavorable attributes while simultaneously providing visual appeal and probiotic health benefits in food products. Various bacterial pigments, such as *β*-carotene, anthocyanidin, riboflavin, and violacein, show potential as food-grade additives.

*β*-Carotene functions as the precursor of provitamin A, a vital fat-soluble nutrient essential for maintaining normal metabolism and physiological functions in the human body. In the food industry, it serves as a colorant added to dairy products, canned fruits, jams, confectioneries, and beverages. Moreover, *β*-carotene is frequently adopted as a nutritional supplement in healthcare products. The utilization of microbial fermentation for carotene production has proven to be superior to plant-based extraction in terms of quality, cost-effectiveness, and technical feasibility. Venil *et al*. achieved a groundbreaking application of spray drying violet pigment from *Chromobacterium violaceum*. Yogurts and jellies colored with this powder colorant produced vivid violet foods, maintaining their color for a month of storage [74]. Additionally, bacterial pigments, such as yellow zeaxanthin derived from *Flavobacterium* spp., possessing antioxidant properties, find application in feed for certain poultry species in laying and fattening processes [27]. Astaxanthin, a common additive in poultry feed, promotes sustained muscle tissue growth in hens and chickens.

### Bacterial Pigments in Industrial Biotechnology

Bacterial pigments have gained attention in industries due to their diverse properties. For example, some photosynthetic bacteria produce chlorophyll. The study reveals that when applied to solar cells, chlorophyll remains effective even under cloudy conditions. Similarly, this pigment can be applied to light-emitting diodes and lasers [75]. Indigo exhibits promising application potential in organic semiconductors, enabling the realization of ambipolar devices [76]. Pigments such as astaxanthin, lycopene, and *β*-carotene, known for their antioxidant properties, are used in cosmetics to resist ultraviolet (UV) light and combat skin aging. External environmental factors such as pollution, UV exposure, and radiation can cause premature skin aging. Carotenoids, with excellent antioxidant properties, prevent the production of reactive oxygen species causing cellular damage. Therefore, they are utilized in anti-aging formulas in face creams. Astaxanthin, reported from *Haematococcus pluvialis*, possesses outstanding antioxidant properties, scavenging cellular free radicals and effectively slowing down the aging process [77]. It has also been found to reduce melanin production and fade skin spots associated with aging [78]. Bacterial pigments serve as additives in cosmetics for their UV-protective properties and can be used as preservatives, being non-toxic and stable, imparting a long shelf-life to the product.

The textile industry can benefit significantly from bacterial pigments. The use of synthetic pigments as colorants may pose various risks to human health, such as allergenicity and the release of potentially harmful compounds during synthesis. Indigo is the primary dye used for the production of cotton denim fabrics and jeans and is applied to more than 4 billion denim garments each year. Employing recombinant bacteria to produce indigo could establish a more sustainable and environmentally friendly manufacturing platform. Natural pigments such as prodigiosins, extracted from *Vibrio* sp., can be utilized for dyeing a variety of fabrics, including silk, wool, acrylics, cotton, and nylon [79]. These pigments offer stable and vibrant colors unaffected by external factors. Similarly, violacein, produced by the marine bacterium *Pseudoalteromonas*, can be used as a fabric dye. Remarkably, these bacteria perish at high temperatures, displaying intolerance even to human temperatures, indicating their safety for industrial purposes.

### Bacterial Pigments in Environmental Biotechnology

Bacterial pigments offer great potential for environmental remediation. Generally, the preference for environmentally friendly and efficient biological agents over chemical agents is evident. Bacterial pigments can contribute to this preference. For instance, *Bacillus thuringiensis* produces an insecticidal crystal protein. However, this crystalline protein is unstable and susceptible to destruction by UV radiation. To safeguard the insecticidal crystal protein, a melanin-producing mutant can be constructed, expressing high levels of UV light-protecting pigment, melanin. Such a mutant could be valuable for the industrial-scale production of light-stable, environmentally friendly insecticides [80]. Similarly, insecticides containing pigments such as violacein can aid in preventing plant mycosis and parasitic nematode diseases. Experiments indicate that prodigiosin is effective in preventing many insects, including *Anopheles stephensi*, *Drosophila larvae*, and *Aedes aegypti* [72, 81, 82].

Bacterial pigments also find application in bioremediation, addressing the issue of heavy metal contaminants in water. For instance, melanogenic bacteria such as the marine bacteria *Pseudomonas stutzeri* synthesize melanin, which can effectively remove heavy metals from water. Melanin nanoparticles can also effectively adsorb heavy metals such as Cu (II), Hg (II), Cr (VI), and Pb (II) from water, making them suitable for sewage treatment [83]. This method proves cost-effective and environmentally friendly compared with traditional physicochemical methods. Additionally, melanin's electron shuttling and metal sequestration capacities make it useful for immobilizing metals and radionuclides such as uranium present in soil [84]. Certain pigmented bacteria can serve as bioindicators monitoring environmental health through the production and/or alteration of specific pigments. For example, the carotenoid content in *Lecanoraceae lichens* depends on atmospheric pollution levels in their environment. Monitoring carotenoid content can aid in assessing the degree of environmental pollution [85]. The production of indigoidine by *Vogesella indigofera* is suppressed by Cr^6+^ in a concentration-dependent manner, serving as an indicator of chromium concentration and toxicity in the environment [86]. Furthermore, bacterial pigments can function as biofertilizers. Some carotenoids, combined with plant growth-promoting substances, can help plants withstand environmental stress and improve their survival rate.

## Advances in Bacterial Pigment Production

**Fig. 4:**
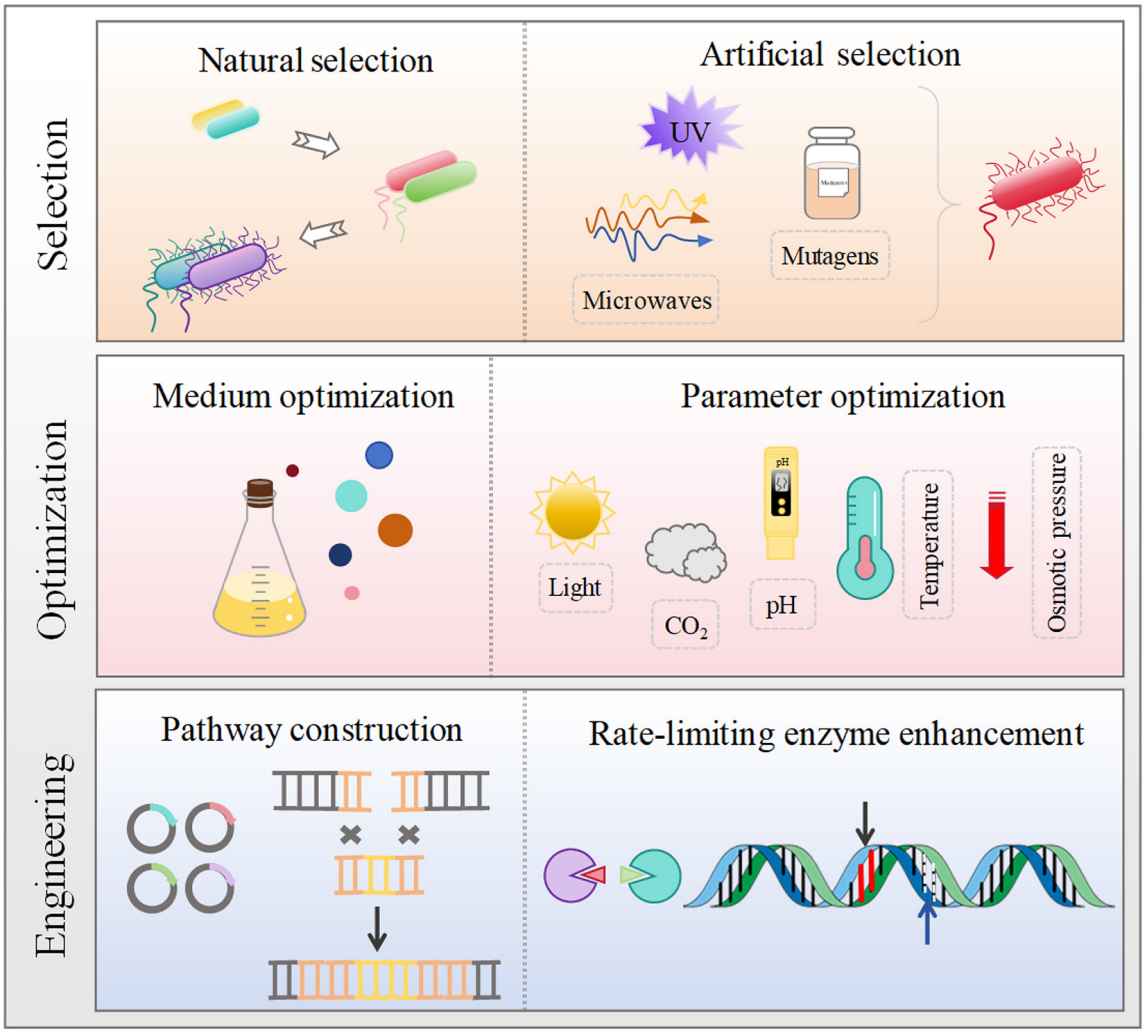
Available strategies for improving the production of bacterial pigments.

Although the commercial development of bacterial pigments has certain advantages, there are still several challenges that need to be overcome when it comes to achieving large-scale production. These encompass issues like low pigment production, unstable quality, and high production costs. Overcoming these challenges requires the development of more efficient production strategies. One approach involves screening and identifying high-yielding strains of bacteria capable of producing the desired pigments. Additionally, optimizing production conditions, including nutrient availability, pH, temperature, and oxygen levels, can significantly enhance pigment production. Moreover, modern genetic engineering techniques can be employed to cultivate bacterial strains with improved pigment synthesis capabilities. This may include manipulating metabolic pathways or introducing genes responsible for pigment production to augment yields and stability. Addressing these challenges and implementing efficient production strategies can lead to the improvement of large-scale bacterial pigment production, rendering it more economically viable and environmentally sustainable. Some available strategies for improving the pigment production by bacterial strains are shown in Fig. 4.

### Natural or Artificial Selection of Pigment Hyperproducer Strains

In general, wild-type strains tend to produce insufficient quantities of biopigments, posing a hindrance to large-scale pigment generation in the industry. Conversely, mutant strains, particularly in the case of *P. aeruginosa*, exhibit the capacity to produce higher amounts of various pigments, such as pyomelanin, pyocyanin, pyoverdin, and pyorubin, in their environmental niches. In the context of *P. aeruginosa* isolates from patients with cystic fibrosis, these mutant strains have demonstrated increased competitiveness compared to their wild-type parents [87].

Genetic engineering techniques have proven effective in screening and identifying mutant strains with high pigment yields. Mutagens such as ethyl methane sulfonate, UV radiation, 1-methyl-3-nitro-1-nitrosoguanidine, and microwaves can be utilized to increase pigment production. For example, inactivating the hmgA gene through mutagenesis is a viable strategy. In the model microorganism *P. aeruginosa* PAO1, a transposon bank can be constructed, and pigment-producing mutants can be screened from it. These methods facilitate the selection and optimization of strains capable of producing larger quantities of desired pigments [88].

### Optimization of Culture Conditions for Native Producers

Optimal production of microbial pigments depends on the precise control of various factors, including pH, osmotic pressure, salinity, temperature, medium nutrition, and light intensity. By carefully adjusting the nutrient composition and environmental parameters of the medium, it becomes possible to enhance the yield of bacterial pigments.

The growth of microorganisms and subsequent pigment synthesis is influenced by the nutrient content of the medium. Manipulating the carbon/nitrogen (C/N) ratio has been demonstrated to regulate pigment production. For instance, Pandey *et al*. discovered that adding maltose as a carbon source in potato glucose broth promoted orange pigment production in certain *Penicillium* strains, while lactose supplementation inhibited this production. Additional nitrogen sources did not promote pigment production, but the addition of mineral salts such as MgSO_4_ and KH_2_PO_4_ enhanced pigment production. Furthermore, the addition of sodium nitrate at a concentration of 0.1 g/l in the media increased carotenoid production in *Arthrospira platensis* [89, 90]. Different nitrogen sources (NaNO_3_, NaNO_2_, NH_4_Cl, and CH_4_N_2_O) had varying effects on the lutein content of *Prochlorococcus* sp., with the highest yield (3.34 mg/g DW) achieved when urea (CH_4_N_2_O) was added to the medium [91]. In Nostoc, the highest phycocyanin content was obtained by supplementing the medium with glucose (0.5 g/l) and nitrate (2 mM) [92].

The pH and temperature of the medium are crucial for bacterial pigment biosynthesis, influencing cellular metabolism and enzyme activity. For example, the Antarctic psychrophilic bacterium *Micrococcus roseus* exhibits high carotenoid production at 5°C but not at elevated temperatures. *M. tuberculosis*, under acidic stress (pH 5–6), produces carotenoid pigments distinct from those of other mycobacterial species [93]. Additionally, light exposure affects pigment production, as certain bacteria such as *Dacryopinax spathularia*, *Myxococcus xanthus*, *Mycobacterium marinum*, and *Rhodotorula glutinis* produce carotenoids in response to light stimuli [94].

The selection of substrates and the techniques employed for separation and purification are crucial factors that significantly influence the cost of fermentative microbial pigment production. In industrial settings, the production of melanin requires the addition of tyrosine, while indigo production necessitates the addition of indole. Both additions contribute to increased production costs. The optimization of substrates can facilitate efficient and cost-effective pigment production. One effective approach involves utilizing agro-industrial residues as raw materials for pigment production, not only converting waste into valuable resources but also reducing production costs.

### Genetic and Metabolic Engineering Approaches for Improved Productivity

The use of microorganisms for pigment production offers several advantages over alternative methods, including fast transformation and the ability to easily adjust the microbial cell factory for high yields. Anthocyanins, which are water-soluble natural pigments imparting various colors to plants under different conditions, can also be produced through microbial fermentation. However, traditional fermentation methods are expensive and yield low results. Recent experiments have shown that efficient anthocyanin production in *Saccharomyces cerevisiae* can be achieved by introducing anthocyanin transporters and knocking out the identified anthocyanin-degrading enzyme [95]. Additionally, pathway reconstruction in microorganisms provides the possibility of low-cost production of natural pigments.

Another common technique involves obtaining high expression of specific genes using expression plasmids. For instance, Maj *et al*. successfully transformed strains of *Paracoccus* sp. into effective producers of xanthophylls and carotenes by creating in vivo plasmids [96]. Similarly, Furubayashi *et al*. increased microbial capsaicin production using a comparable approach. Capsaicin is a stimulant carotenoid commonly found in *Capsicum annuum*. They engineered a heterologous capsanthin biosynthetic pathway in *Escherichia coli* by expressing eight genes, including five zeaxanthin biosynthesis genes from a soil bacterium (*Pantoea ananatis*), zeaxanthin epoxidase, capsanthin/capsorubin synthase from *Capsicum annuum*, and isopentenyl diphosphate isomerase from green alga (*Haematococcus pluvialis*). After critical upregulation of carotenogenic genes and minimizing by-product formation, they achieved a production level of 0.5 mg/l capsanthin [97].

In summary, these research advancements have enhanced the efficiency of bacterial pigment production at the laboratory scale and accelerated the commercialization of microbial pigment production. Nevertheless, some technologies are not yet mature enough for large-scale production of microbial pigments. Therefore, it is crucial to reduce production costs and improve separation and purification technologies to facilitate the widespread application of microbial pigments.

## Conclusion and Future Prospects

Currently, the trend toward utilizing microorganisms for the production of natural pigments is undeniable, and the research focus has shifted toward multifunctional bacterial pigments. Notably, model microorganisms such as *E. coli* and *S. cerevisiae* have made significant advancements in pigment development because of their well-defined features and genetic background [98]. With the right strategy, bacterial pigments have the potential to replace all synthetic pigments currently used in the industry.

In recent years, significant strides have been taken in studying the isolation of pigment-producing microorganisms in the ocean and cryosphere. Pigmented bacteria within these niches represent an untapped treasure trove. Exploring pigment-producing bacteria in these environments is poised to yield a valuable resource for novel pigment molecules with broader biotechnological applications. The screening of pigment-producing bacteria with specific biological activities and incorporating them into drugs, cosmetics, and food products has the potential to revolutionize our lifestyle and safeguard our health. In the future, the substitution of more natural pigments for synthetic ones will not only enhance pigment production efficiency but also reduce costs, paving the way for the gradual universal adoption of bacterial pigments.

## Figures and Tables

**Table 1:** Some common bacterial pigments and their biological sources, applications and structures.